# Sacral Chordoma Presenting as Back Pain in the Chiropractic Clinic: A Case Report

**DOI:** 10.7759/cureus.39810

**Published:** 2023-05-31

**Authors:** Aaron Ka-Chun Chan, Gabriel Siu Nam Ng, Benjamin Kah Chun Cheong, Kenny Kim Pong Ng, Eric Chun-Pu Chu

**Affiliations:** 1 Chiropractic and Physiotherapy Centre, New York Medical Group, Hong Kong, CHN; 2 Medical Oncology, reVIVE Oncology and Cancer Centre, Hong Kong, CHN

**Keywords:** back pain, chiropractor, gluteal pain, lumbar pain, chiropractic, sacral chordoma

## Abstract

Cases of lumbar and gluteal pain are commonly encountered in chiropractic clinics, with a broad differential diagnosis primarily centered on musculoskeletal conditions. This report presents the second documented case of sacral chordoma diagnosed at a chiropractic clinic and emphasizes the importance of considering alternative diagnoses and interdisciplinary collaboration in patient care. A 42-year-old man presented to a chiropractic clinic with complaints of lumbar and gluteal pain. The initial conservative management based on a presumptive musculoskeletal diagnosis was ineffective. Suspicion of an alternative etiology prompted a referral for imaging, which revealed a sacral chordoma. An interdisciplinary collaboration involving orthopedic surgeons, oncologists, radiologists, and other healthcare professionals was initiated to optimize the treatment outcomes of this rare and aggressive tumor. This case report underscores the importance of maintaining a high index of suspicion in cases of musculoskeletal presentations in chiropractic clinics and the critical role of advanced imaging in establishing a definitive diagnosis. Interdisciplinary collaboration is essential for managing complex conditions such as sacral chordomas, ensuring the delivery of the highest quality of care, and optimizing patient outcomes. Chiropractors play a crucial role in identifying, referring, and contributing to the management of patients with complex presentations as part of a comprehensive multidisciplinary treatment plan.

## Introduction

Chordomas are rare malignant bone tumors originating from embryonic remnants of the notochord and account for approximately 1-4% of all primary bone malignancies [1]. These slow-growing neoplasms predominantly affect the axial skeleton, with approximately 50-60% of tumors occurring in the sacrococcygeal region [2]. Patients with sacral chordomas often present with insidious, nonspecific symptoms caused by the compression and/or destruction of adjacent structures by the tumor [3]. The clinical course of sacral chordomas is generally indolent, but these tumors possess a notable propensity for local recurrence and metastatic potential, necessitating prompt diagnosis and appropriate management [4]. Approximately one-third of patients with sacral chordomas also present with urinary tract infections, constipation, or disc herniation symptoms. The most prevalent presenting symptom in patients with sacral chordomas is sitting-aggravated low back pain [3]. This overlap in clinical features can pose diagnostic challenges for healthcare providers, including chiropractors, who frequently encounter patients with musculoskeletal disorders. Thorough clinical examinations and consideration of differential diagnoses are crucial for guiding appropriate imaging and diagnostic workups. In cases where the clinical presentation is refractory to conservative management, advanced imaging techniques, such as magnetic resonance imaging (MRI), are warranted to elucidate the underlying etiology.

In the present case report, we describe the diagnosis and management of a sacral chordoma in a patient who visited a chiropractic clinic with radiating left gluteal pain. To our knowledge, this is only the second case diagnosed in a chiropractic clinic in the literature, according to a search conducted on May 18, 2023, using the databases PubMed, Index to Chiropractic Literature, and Google Scholar [5]. By detailing the clinical course, diagnostic process, and interdisciplinary collaboration in this case, our goal is to emphasize the importance of considering alternative diagnoses and using advanced imaging to manage complex presentations, particularly in cases of lumbar and gluteal pain, and to stress the importance of prompt referral to specialist care.

## Case presentation

A 42-year-old man presented to our chiropractic clinic with a two-week history of left gluteal pain following a fall on his buttocks. The patient described the onset of symptoms as moderate coccygeal pain that gradually improved, followed by worsening left gluteal pain radiating to the posterior left thigh. The pain was exacerbated by supine positioning and prolonged sedentation and relieved by ambulation and changing positions. The patient denied having hypoesthesia, asthenia, paresthesia, or other radicular symptoms. The patient reported chronic lumbosacral tension and rigidity. The patient’s medical history was noteworthy for a similar episode of left gluteal pain that occurred 30 years ago and persisted for 12 months. He recently sought treatment from a traditional Chinese medical practitioner involving an unspecified bone setting, which resulted in mild relief of current symptoms. Ten years ago, the patient underwent a right knee meniscectomy and tendon repair for meniscal and tendinous lacerations. No history of any other chronic illnesses, hospitalizations, trauma, surgeries, or medication use was present. The patient reported a history of recurrent lumbosacral and gluteal pain, intermittently managed with various oral analgesics and nonsteroidal anti-inflammatory medications over the past two weeks. He initially consulted an orthopedist who prescribed two weeks of rest and home cryotherapy. However, these measures provided only minimal relief from the symptoms. He then sought chiropractic care for the gluteal pain.

**Figure 1:**
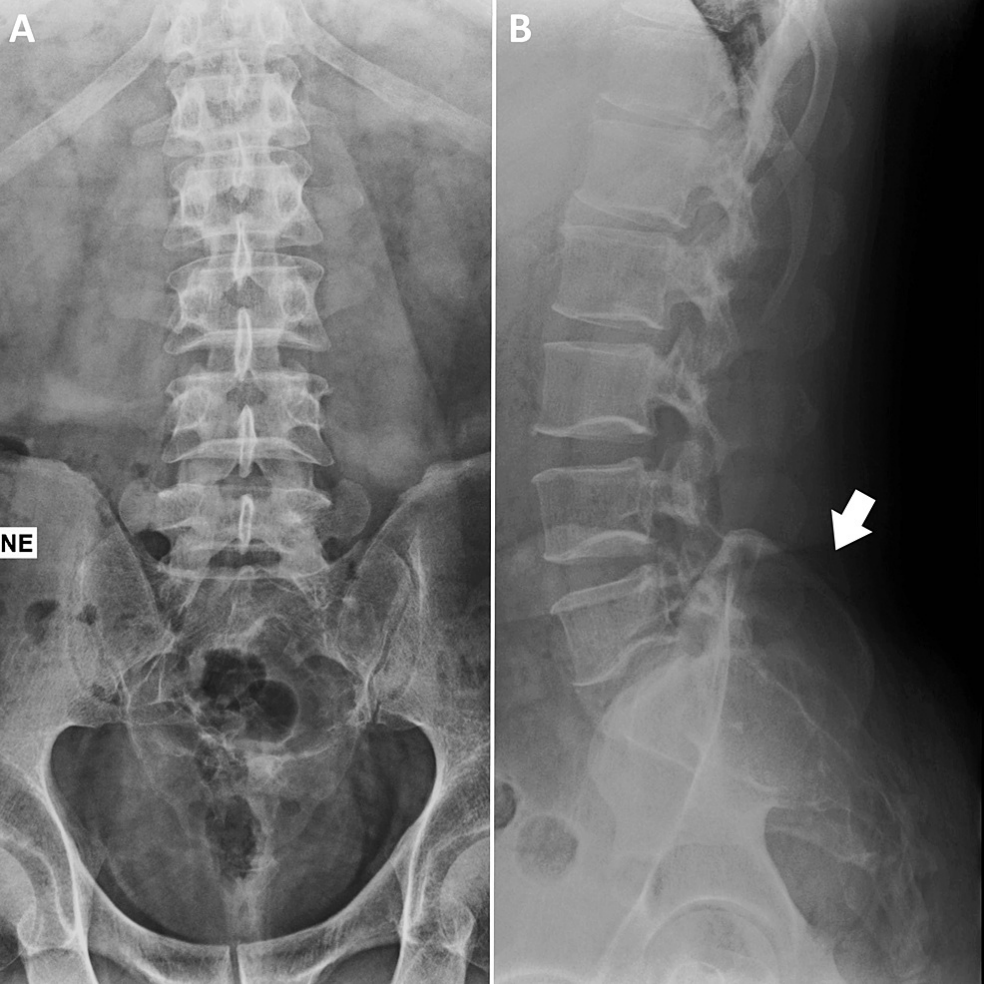
Lumbar radiographs. An expansile lytic lesion is identified at the sacrum (white arrow) in both (A) anterior-posterior and (B) lateral views of the lumbar radiographs.

The patient did not have any unexplained weight loss, fatigue, fever, or other constitutional symptoms. His occupation as an administrative assistant involved prolonged sitting. He exercised thrice weekly and swam for 30 minutes on alternate days. Physical examination by the chiropractor revealed that the patient had no acute distress; the pain was rated 5/10 on a numeric scale, and the quality of life was scored at 84%. The active range of motion of the thoracolumbar spine was full with no pain. Palpation of the lumbosacral region elicited paraspinal tenderness and induration, most prominently at the L5-S1 level. Although the patient reported having no radicular symptoms, the straight leg raise testing of the left lower limb reproduced the patient’s gluteal and posterior thigh symptoms at 60 degrees. Deep tendon reflexes were bilaterally equal at 2+ with downward halluces. Manual muscle testing revealed no focal deficits in the major muscle groups of the lower extremities. Overall, the orthopedic examination revealed lumbosacral paraspinal muscular spasming and tenderness and reproduction of radiating pain in the left lower limb during the straight leg raising test, consistent with compression of the existing left first sacral nerve root. The neurological assessment revealed intact motor function, sensations, and symmetrical reflexes, without evidence of upper motor neuron damage. Owing to worsening symptoms recalcitrant to two weeks of rest, a lumbar radiographic investigation was influenced by the patient’s concerns and request, which revealed an expansile lytic lesion at the sacrum (Figure 1).

**Figure 2:**
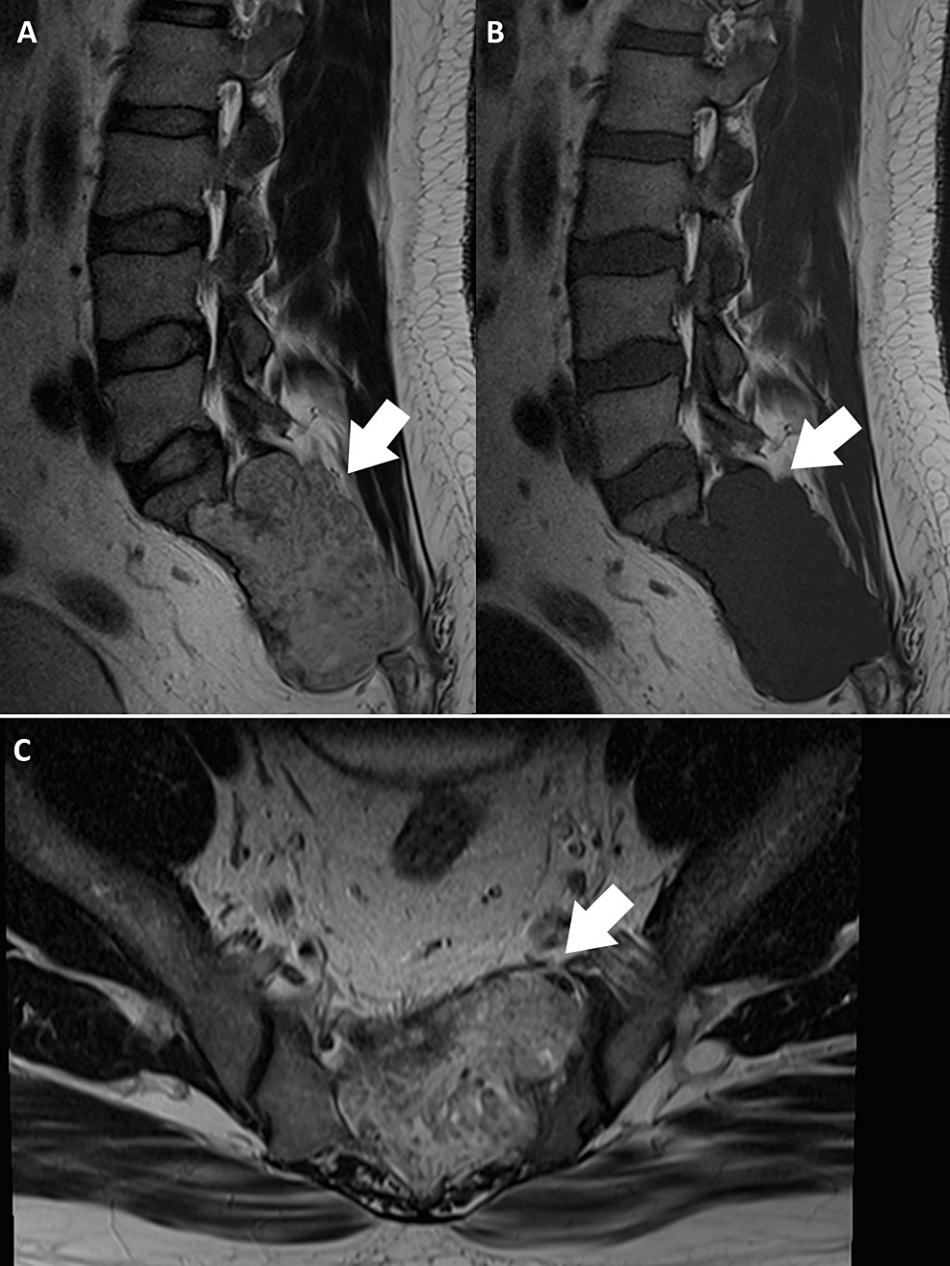
Lumbar magnetic resonance image (MRI): The presence of a moderately hypermetabolic soft tissue mass involving S1-S3 vertebral bodies with a presacral soft tissue component and measuring 6.96 × 5.12 cm (white arrow) strongly suggests a malignant process. (A) On T2-weighted images, high signal intensity can be seen. Destruction or erosion of the sacral bone, and possible extension into adjacent structures, can also be identified. (B) On T1-weighted images, the image appears isointense compared to the normal spinal cord. (C) On the coronal view, destruction or erosion of the sacral bone can be seen.

**Figure 3:**
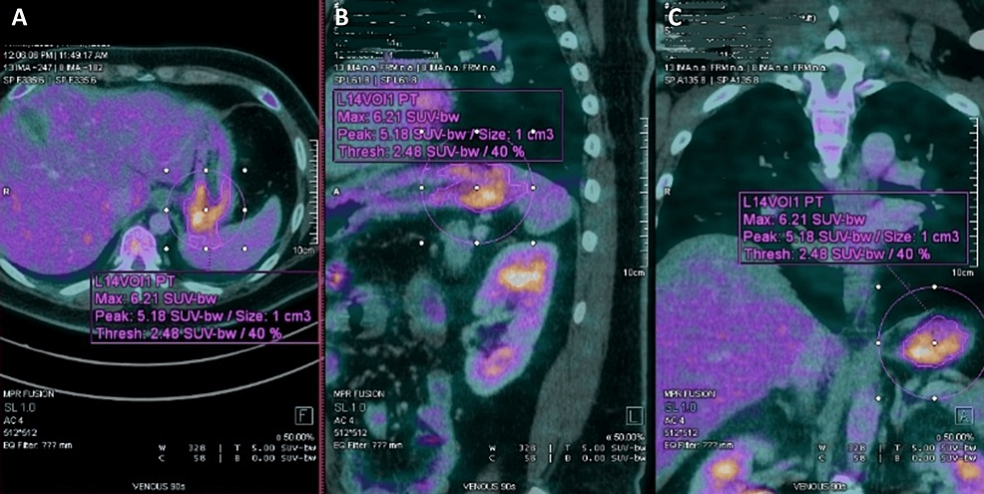
Whole-body positron emission tomography-computed tomography (PET-CT) excluding the brain. A presacral soft tissue component and a moderately hypermetabolic soft tissue mass (circle) involving the S1–S3 vertebral bodies can be observed (SUVmax: 6.16) at the coronal (A), lateral (B) and anterior-posterior views (C), which is strongly suggestive of a malignant process.

An advanced lumbar MRI was performed to further investigate the cause of localized and radiating pain. The MRI revealed a lobulated lesion with T1 hypointensity and T2 hyperintensity spanning the S1-S3 vertebral corpora, considerably stenosing the canal centrally and compressing the thecal sac (Figure 2). In addition, severe focal narrowing of the left S1 foramen with compression of the existing S1 root was noted. Signs of rectal infiltration or enlarged pelvic lymphadenopathy were absent. Based on the clinical presentation and MRI findings, the patient was diagnosed with sacral chordoma. Given the neoplastic features, including the size of the tumor, canal stenosis, and invasion into the surrounding soft tissues, the chiropractor urgently referred the patient for oncological evaluation. The total-body positron emission tomography-computed tomography (PET-CT) revealed localized disease without metastasis (Figure 3). The patient was transferred to a spine surgeon. One week after the orthopedic consultation, the patient reported that he underwent en bloc resection of the sacral mass via a combined anterior-posterior approach with S1-S3 sacrectomy achieving negative margins. He experienced relief from the symptoms and recovered well after the surgery.

## Discussion

The differential diagnoses of conditions causing lumbar and gluteal pain in chiropractic clinics are varied, and the present patient’s medical history highlights the importance of considering alternative diagnoses before initiating treatment. The diagnosis of sacral chordoma typically involves a combination of clinical examination, imaging studies, and biopsy. A patient’s medical history and physical examination may reveal symptoms and signs suggestive of a sacral chordoma. While musculoskeletal conditions are commonly encountered by chiropractors [6], the present case highlights the importance of being vigilant for rare diseases, such as sacral chordomas, which may present with nonspecific clinical features mimicking more common musculoskeletal disorders [5,7-17]. Both patients presented with symptoms suggestive of a sacral chordoma and the MRI findings were consistent with the typical features of a sacral chordoma. Both cases were immediately referred to a spine surgeon after chiropractic consultations. However, unlike the previous case report of chordoma, our patient was immediately diagnosed in the initial consultations [5]. The present case underscores the importance of maintaining a high index of suspicion in cases of musculoskeletal presentations, conducting thorough clinical examinations, and considering appropriate differential diagnoses to guide patient care [18].

Advanced imaging played a crucial role in the diagnostic process in the present case, particularly given the musculoskeletal presentation and rarity of sacral chordomas. Although plain radiographs were initially used as the imaging modality of choice, they were unable to provide sufficient details of the pathology. On T1-weighted images, sacral chordomas usually appear isointense or slightly hypointense from the sacrum and may extend into the surrounding tissues compared to the normal spinal cord. On T2-weighted images, they generally display high signal intensity. Destruction or erosion of the sacral bone, as well as possible extension into adjacent structures, were also identified in our case. Advanced imaging techniques, including MRI, were instrumental in establishing a clear diagnosis [19]. Chiropractors must be proficient in deciding when to refer patients for advanced imaging, as in the present case, and interpreting the results to guide clinical decision-making [20]. Interdisciplinary collaboration was essential for managing this complex case of sacral chordoma. Owing to the rarity and aggressive nature of this tumor, specialized care is required to optimize patient outcomes, necessitating collaboration with orthopedic surgeons, neurosurgeons, oncologists, radiation therapists, and other healthcare professionals [3].

In Hong Kong, chiropractors are trained to diagnose and treat musculoskeletal and neurological conditions [21] and are considered the primary care clinicians who frequently manage low back pain [22]. The present case highlights the crucial role chiropractors play in the collaborative process by identifying and referring patients with complex presentations of low back pain to appropriate specialists promptly. Furthermore, chiropractors can contribute their expertise to the conservative management of pain and functional improvement as part of a comprehensive, multidisciplinary treatment plan. By fostering open communication and collaborating as a team, healthcare providers can ensure that patients receive the highest quality of care and achieve the best possible outcomes.

## Conclusions

This report presents the second documented case of sacral chordoma diagnosed at a chiropractic clinic and emphasizes the importance of considering alternative diagnoses in the differential diagnosis of lumbar and gluteal pain. Chiropractors must maintain a high index of suspicion in cases of musculoskeletal presentations and employ comprehensive clinical examinations to guide patient care. Advanced imaging techniques, such as MRI, provide invaluable insights and can be instrumental in establishing definitive diagnoses of rare and complex pathologies. An interdisciplinary collaboration involving orthopedic surgeons, neurosurgeons, oncologists, radiation therapists, and other healthcare professionals is essential for managing complex conditions such as sacral chordomas. By fostering open communication and collaborating as a team, healthcare providers can ensure the delivery of the highest quality care and optimize patient outcomes despite challenging diagnostic and therapeutic scenarios.
